# Functional Structure of Biological Communities Predicts Ecosystem Multifunctionality

**DOI:** 10.1371/journal.pone.0017476

**Published:** 2011-03-10

**Authors:** David Mouillot, Sébastien Villéger, Michael Scherer-Lorenzen, Norman W. H. Mason

**Affiliations:** 1 Laboratoire Ecologie des Systèmes Marins Côtiers UMR 5119, Université Montpellier 2, Montpellier, France; 2 Laboratoire Evolution et Diversité Biologique UMR 5174, Université Paul Sabatier, Toulouse, France; 3 Faculty of Biology - Geobotany, University of Freiburg, Freiburg, Germany; 4 Landcare Research, Hamilton, New Zealand; Dalhousie University, Canada

## Abstract

The accelerating rate of change in biodiversity patterns, mediated by ever increasing human pressures and global warming, demands a better understanding of the relationship between the structure of biological communities and ecosystem functioning (BEF). Recent investigations suggest that the functional structure of communities, i.e. the composition and diversity of functional traits, is the main driver of ecological processes. However, the predictive power of BEF research is still low, the integration of all components of functional community structure as predictors is still lacking, and the multifunctionality of ecosystems (i.e. rates of multiple processes) must be considered. Here, using a multiple-processes framework from grassland biodiversity experiments, we show that functional identity of species and functional divergence among species, rather than species diversity *per se*, together promote the level of ecosystem multifunctionality with a predictive power of 80%. Our results suggest that primary productivity and decomposition rates, two key ecosystem processes upon which the global carbon cycle depends, are primarily sustained by specialist species, i.e. those that hold specialized combinations of traits and perform particular functions. Contrary to studies focusing on single ecosystem functions and considering species richness as the sole measure of biodiversity, we found a linear and non-saturating effect of the functional structure of communities on ecosystem multifunctionality. Thus, sustaining multiple ecological processes would require focusing on trait dominance and on the degree of community specialization, even in species-rich assemblages.

## Introduction

Ecosystems are facing ever increasing levels of human pressures which imperil the goods and services they provide to humanity. It is now recognized that both changes in environmental conditions (e.g., global warming) and modifications to biological communities (e.g., biodiversity erosion) affect ecosystem processes [1], [2], [3], the latter issue having stimulated convincing advances but also controversy [4]. During the last two decades the positive relationship between biodiversity and ecosystem functioning (BEF hereafter) has been demonstrated through experiments manipulating species composition in model assemblages [2], [4], [5]. These studies helped to place the problems of environmental change and biodiversity loss into the mainstream political agenda [6]. However, there is an urgent need to move beyond the heuristic objective of early biodiversity experiments, and then to disentangle the contributions of the various components of biodiversity on ecosystem processes and, ultimately, to build a predictive framework for BEF research that can forecast the potential effects of biodiversity changes that all ecosystems on earth are experiencing.

To reach this objective, at least two limitations remain. First, biodiversity has been recognized as a multidimensional concept [7], [8] but many BEF studies rely solely on species richness for practical reasons and remain silent on the functional structure of communities. Yet, the functional trait composition of biological communities is a key component that most often explains ecosystem functioning better than species richness *per se* whatever the biota [2], [9], a functional trait being any morphological, physiological or phenological feature, measurable at the individual level, that determines species effects on ecosystem properties [10]. The limited predictive power of BEF research, even if biodiversity effects were demonstrated to be positive and significant [11], [12], [13], is certainly due to the clear initial focus on testing diversity effects (mostly on the species richness level) irrespective of other compositional factors, such as species or functional identity, and the resultant lack of an integrative framework where different components of biodiversity were considered altogether as predictor variables. Second, the vast majority of BEF studies have focused on a single ecosystem process (e.g. productivity) while overall ecosystem functioning is sustained by several processes [14]. Recent results suggest that the effect of biodiversity in natural ecosystems may be much larger than currently thought if we consider a multiple-processes framework [15], .

Taxonomic diversity, functional identity and functional diversity of ecological communities are each known to influence ecosystem processes but their relative effects remain largely untested, particularly in predicting rates of multiple ecosystem processes [18], [19], [20]. Species richness was the first biodiversity component to be related to ecosystem functioning [21] supporting the hypothesis that species complementarity and sampling effects both enhance resource use and productivity. Then, species evenness, or how equitably abundance is distributed among species within a community, was demonstrated to positively influence productivity [22]. The functionally orientated BEF research began as early as 1941 [23] with the study of the effect of particular species functional traits (functional identity) on ecosystem processes (soil formation). Then, other authors have pointed out that ecosystem properties should primarily depend on the identity of dominant species and their functional traits following the ‘mass ratio hypothesis’ [24], [25]. Indeed, functional identity, usually expressed as the biomass-weighted mean trait value for a community, has been demonstrated to be a key driver of ecosystem functioning from local [20] to regional [26] and global [27] scales. Beyond functional identity, functional diversity, defined as the diversity and abundance distribution of traits within a community [28], has been shown to be an accurate predictor of ecosystem functioning [29], [30], [31], reinforcing the importance of niche complementarity for enhancing ecosystem processes [32].

All these biodiversity components are not mutually exclusive but are unlikely to exert equal influence on ecosystem processes and on the multifunctionality of ecosystems. Thus the question is no longer whether each of the three components of biodiversity (taxonomic diversity, functional identity and functional diversity) matters but whether it still matters after removing the effect of two other components? In other words, we examined the additional effect of each biodiversity component on the prediction of ecosystem processes to determine whether each component has an essential and complementary contribution to the explanation of ecosystem multifunctionality. Further, by including the eight biodiversity indices, embracing all aspects of taxonomic and functional structure of communities, we built a minimum adequate model that reached an unprecedented level of explanatory power with functional identity and functional diversity together as predictor variables of multiple ecosystem processes. Finally, we implemented structural equation models to explore both causal and spurious associations between predictors of ecosystem processes.

We used data on several ecosystem processes including biomass production and decomposition trials within the German BIODEPTH experiment (BIODiversity and Ecological Processes in Terrestrial Herbaceous Ecosystems) to predict the effects of biodiversity change on ecosystem functioning. This experiment allows testing all components of biodiversity given that, for each species richness level, different species combinations were constructed.

## Materials and Methods

### Experiment

Data have been collected at the German site of the pan-European BIODEPTH project [33]. A gradient of plant species richness and number of functional groups (grasses, legumes, non-leguminous herbs) was created by sowing mixtures containing 1, 2, 4, 8 or 16 species, typically found in mid-European hay meadows (total species pool was 31 species). Total seed density was 2,000 viable seeds per m^2^, equally divided among all species following a substitutive replacement series design. Each diversity level was replicated with several mixtures differing in species composition. The whole experiment was replicated in a second block with new randomization of plots, yielding a total of 60 plots of 2×2 m in size. Unsown seedlings were continuously weeded, and the plots were not fertilized. Among the 60 plots, we retained only 26 since we had to select only those with species richness ranging from 4 to 16 species to be able to estimate indices of functional community structure. Indeed, there is no functional volume (functional richness index) with 1 or 2 species. As an alternative, we should use the Functional Diversity (FD) index [34], based on a dendrogram, to cope with species poor communities (less than 3 species) and thus use the entire range of species richness available in BIODEPTH. However, the building of functional dendrograms is contentious [35] and we cannot estimate the other functional diversity components (those including species abundances) with this approach.

### Ecosystem processes

Among the ecological processes that were measured at the German BIODEPTH site we selected those that were relatively independent (mean correlation over all selected processes was 0.5) since two highly correlated processes would be trivially ruled by the same biodiversity components. The response variables were cotton decomposition in 1997 and 1998, litter decomposition in 1998, productivity in 1997 and 1998, and nitrogen pool size in aboveground biomass 1998. Cotton decomposition trials are a standard method to test for effects of microenvironmental conditions on decomposition processes. It was measured as dry weight loss (g.g^−1^.d^−1^) of a standard cotton fabric using strips of 5×12 cm (Shirley Soil Burial Test Fabric, c. 95% cellulose; initial nitrogen concentration of 0.09%) during 10 weeks of field exposure in all experimental communities, with three strips per plot [31], [36]. Litter decomposition was the dry weight loss (g.g^−1^.d^−1^) of plot-specific senescent leaf and stem material, sealed in litter bags of 5×5 cm made of a 0.5 mm nylon mesh, during 10 weeks in autumn 1998.

Macrofauna was excluded with this mesh size. Assuming an equal effect of a small mesh size in all treatments, excluding one decomposer group should not have an effect on our results. This assumption might not be true in case of a diversity effect on macrofauna occurrence. In another experiment, carried out on the same plots and half a year later, we could show, however, that several indices of soil fauna, including different groups of earthworms (litter feeding epigeics and anecics) and nematodes, were not influenced by our plant diversity treatments [37].

The litter bags were placed in a homogeneous patch of an adjacent meadow, thus quantifying the effect of community-specific litter composition and quality on decomposition processes, independent of differences in microenvironmental conditions induced by the experimental communities. Thus, both decomposition trials independently quantified different pathways of potential diversity effects on decomposition processes [31]. Productivity was the sum of two harvests per year (June and September) in 1997 and 1998, as a proxy for annual biomass production (dry weight, g.m^−2^). Standing biomass was cut at a height of 5 cm in two areas of 0.5 m×0.2 m each within a permanent quadrat placed in the center of each plot [33], [36]. Nitrogen pool size in aboveground biomass 1998 was the nitrogen content in dried aboveground biomass of the year 1998, calculated as the product of N concentration and biomass (gN.m^−2^). Nitrogen was measured by dry combustion with an automated C/N analyzer (Carlo Erba NA 1500, Carlo Erba, Mailand, Italy) [33], [36]. We also calculated a multifunctionality variable as the mean performance of communities over the four processes after standardizing each community performance (mean of 0 and standard deviation of 1) in order to give them the same weight. When the same process was measured for two years we first calculated a mean value for this process over the two years.

### Functional traits

The selected traits were growth form: caespitosa, reptantia, scandentia, semirosulata and rosulata; leaf size: nanophyllous (20–200 mm^2^), microphyllous (2–6 cm^2^), submicrophyllous (6–20 cm^2^) and mesophyllous (20–100 cm^2^); leaf seasonality: summergreen, partly evergreen and evergreen, CN ratio of plant litter [31]; SLA based on measurements in another biodiversity experiment [38]; and leaf angle: predominantly vertical leaf orientation, predominantly inclined leaf orientation and predominantly horizontal leaf orientation [38]. See Table S1 for details by species.

### Indices for community structure

We considered two independent variables related to taxonomic composition: species richness and the evenness of abundance distribution among species using the Pielou index [39]. Since we have both quantitative and qualitative traits, we performed a Principal Coordinate Analysis (PCoA) on a Gower distance matrix to provide three independent axes that summarize species distribution within a trait functional space [39]. The functional structure of each community was assessed within this 3-dimensional PCoA space which represents more than 90% of the total inertia. These three independent functional axes from PCoA were used to measure functional identity through biomass-weighted mean trait values for each community.

**Figure 1:**
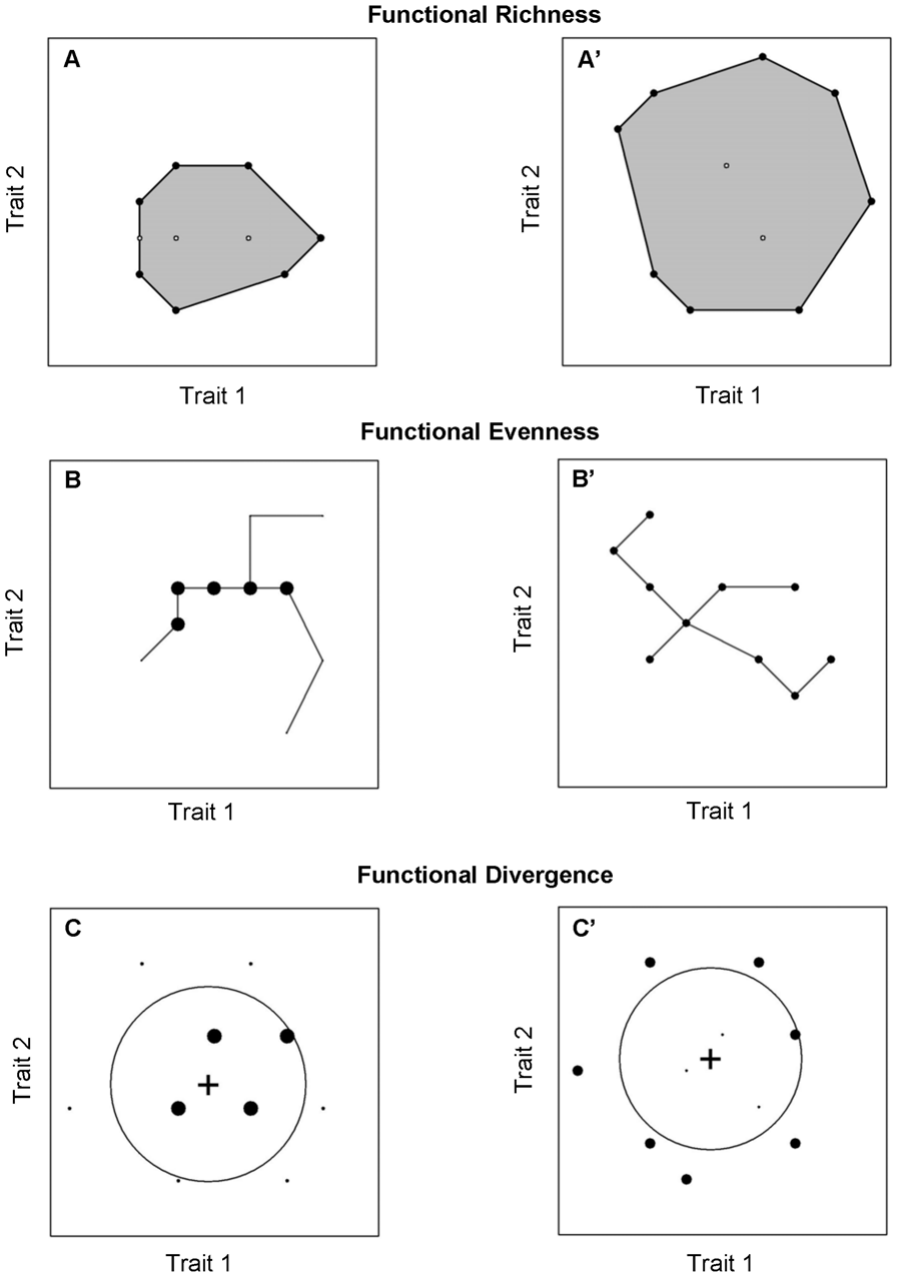
Geometrical presentation of functional diversity indices. For simplicity, only two traits are considered to define a two-dimensional functional space. For the 6 panels, a local community of 10 species (dark disks) is considered among a regional pool of 25 species (grey crosses). Species are plotted in this space according to their respective trait values while the circle areas are proportional to their abundances. Functional diversity of a community is thus the distribution of species and of their abundances in this functional space. Functional richness is the functional space occupied by the community, functional evenness is the regularity in the distribution of species abundances in the functional space and functional divergence quantifies how species abundances diverge from the centre of the functional space. For each component of functional diversity, two contrasting communities are represented, the right column showing an increase of the index value. More details on indices can be found in Text S1.

Three independent variables were related to functional diversity [40] (Figure 1). Functional richness was measured as the amount of functional space filled by the community which is the volume inside the convex hull that contains all trait combinations represented in the community, which basically corresponds to a multivariate functional range [40], [41]. Functional evenness was estimated as the regularity of abundance distribution in the multidimensional functional space, i.e. the regularity with which species abundances fill the functional space. Finally, functional divergence quantified whether higher abundances are close to the volume borders, i.e. whether specialist species *sensu* Elton [42] have the highest abundances. See Text S1 for details on functional diversity indices.

### Statistical analyses

In order to disentangle the relative effect of each biodiversity component on ecosystem processes, several alternative nested models were tested. We used the generalized likelihood ratio test [43] to determine whether each biodiversity component has a significant additional contribution to the explanation of ecosystem processes. Then the parsimony of each model was assessed using the AICc criteria given the ratio between the number of observations (26) and the number of variables (8) [43].

In order to prioritize the biodiversity indices related to ecosystem processes, and to investigate their effects (coefficients), we followed a multiple regression approach. Starting with a full model including all 8 indices, the relative importance of indices was assessed using a backward selection procedure. The significance of the increase in deviance resulting from the deletion of a variable in the model was estimated using the chi-squared deletion test [44]. The minimal adequate model was selected as the one containing nothing but significant variables. For each response variable (ecosystem process), we performed multiple regressions and we then selected the minimal adequate model. We did not rely on classical AIC, BIC or AICc criteria to select the most parsimonious model, i.e. the one offering an optimal trade-off between increased information (number of explicative variables) and decreased reliability (goodness-of-fit), since the number of potential models with 8 predictors vastly exceeds the number of observations [44]. This may lead to spurious model selection results [43].

To correctly estimate the influence of each biodiversity index on ecosystem processes we need to rely on independent biodiversity predictors, since the inherent collinearity among explanatory variables has blurred many statistical and inferential interpretations in ecology [45]. This potential multicollinearity among predictive variables was tested using the variance inflation factor (VIF) [46].

However, even if VIF values are lower than 10, we may still obtain significant biases in parameter estimates and low statistical power, potentially impairing the identification of significant effects and invalidating approaches assuming no collinearity among predictor variables [45]. To examine the role of co-varying factors, we constructed and applied structural equation models (SEMs) for each ecosystem process. This allows direct and indirect effects of the variables of interest to be teased apart and has already applied in BEF research [20]. On one hand, taxonomic diversity or species composition may have significant effects on ecosystem processes but they should be driven by relationships with functional community structure [4]. On the other hand, taxonomic diversity is not expected to be perfectly correlated with functional structure [37]. SEMs allow us to test simultaneously how well functional structure accounts for any effects of taxonomic diversity on EF, and how strongly taxonomic diversity influences functional structure. This will ultimately provide a causal framework linking taxonomic diversity and EF via functional community structure.

All statistical analyses were carried out using R software and packages ‘qpcR’, ‘car’ and ‘lavaan’.

## Results

### Contribution of each biodiversity component

First we ran four linear models for each ecosystem process: the full model including taxonomic diversity (TD), functional identity (FI) and functional diversity (FD) with 8 biodiversity indices (TD+FI+FD), the model without any taxonomic component (FI+FD) where species richness and evenness were removed, the model without any functional identity component (TD+FD) where the biomass-weighted mean trait values were removed, and the model without any functional diversity component (TD+FI) where the 3 functional diversity indices were removed. The most parsimonious model, according to the AICc criteria, was the model without any taxonomic component for all processes but litter decomposition (Table 1). This FI+FD model also provided the highest adjusted *R^2^* values whatever the process except litter decomposition and productivity in 1997 (Table 1).

**Table 1:** Summary of model comparisons for each ecosystem process as well as multifunctionality.

Process	Model	*df*	*AICc*	*R^2^*	*p*	Test	*L.Ratio*	*p*
Cottondecom 97	TD+FI+FD	16	−216.9	0.25	0.114			
	FI+FD	18	**−227.0**	**0.313**	0.041	TD+FI+FD vs. FI+FD	0.243	0.787
	TD+FD	19	−226.9	0.225	0.076	TD+FI+FD vs. TD+FD	1.205	0.340
	TD+FI	19	−222.1	0.059	0.306	TD+FI+FD vs. TD+FI	2.612	**0.087**
Cottondecom 98	TD+FI+FD	17	−229.8	0.29	0.073			
	FI+FD	19	**−239.5**	**0.354**	0.022	TD+FI+FD vs. FI+FD	0.149	0.863
	TD+FD	20	−238.8	0.256	0.049	TD+FI+FD vs. TD+FD	1.320	0.301
	TD+FI	20	−228.2	0.119	0.794	TD+FI+FD vs. TD+FI	4.838	**0.013**
Litterdecom	TD+FI+FD	17	−291.9	**0.648**	<0.001			
	FI+FD	19	−295.8	0.599	<0.001	TD+FI+FD vs. FI+FD	2.317	0.129
	TD+FD	20	−297.0	0.572	<0.001	TD+FI+FD vs. TD+FD	2.436	0.100
	TD+FI	20	**−298.1**	0.589	<0.001	TD+FI+FD vs. TD+FI	2.124	0.135
Productivity 97	TD+FI+FD	17	368.1	**0.794**	<0.001			
	FI+FD	19	**361.2**	0.791	<0.001	TD+FI+FD vs. FI+FD	1.139	0.344
	TD+FD	20	367.6	0.701	<0.001	TD+FI+FD vs. TD+FD	4.030	**0.025**
	TD+FI	20	362.9	0.751	<0.001	TD+FI+FD vs. TD+FI	2.418	0.102
Productivity 98	TD+FI+FD	17	367.0	0.713	<0.001			
	FI+FD	19	**358.4**	**0.725**	<0.001	TD+FI+FD vs. FI+FD	0.594	0.563
	TD+FD	20	358.6	0.695	<0.001	TD+FI+FD vs. TD+FD	1.413	0.273
	TD+FI	20	367.5	0.567	<0.001	TD+FI+FD vs. TD+FI	4.381	**0.019**
Npool bm 98	TD+FI+FD	17	131.3	0.823	<0.001			
	FI+FD	19	**122.2**	**0.834**	<0.001	TD+FI+FD vs. FI+FD	0.363	0.701
	TD+FD	20	127.8	0.77	<0.001	TD+FI+FD vs. TD+FD	2.980	**0.061**
	TD+FI	20	134.9	0.698	<0.001	TD+FI+FD vs. TD+FI	5.689	**0.007**
Multifunctionality	TD+FI+FD	17	51.3	0.751	<0.001			
	FI+FD	19	**42.8**	**0.762**	<0.001	TD+FI+FD vs. FI+FD	0.552	0.586
	TD+FD	20	47.7	0.679	<0.001	TD+FI+FD vs. TD+FD	2.916	**0.064**
	TD+FI	20	52.1	0.62	<0.001	TD+FI+FD vs. TD+FI	4.495	**0.017**

The weight of support for the alternative models (TD: taxonomic diversity, FI: functional identity, FD: functional diversity) and estimates of model parameters for each ecosystem process (Cottondecomp: cotton decomposition, Litterdecom 98: litter decomposition in 1998, Productivity: productivity as annual biomass production, Npool bm: nitrogen pool size in aboveground biomass, Multifunctionality: mean performance over all processes). Results of likelihood ratio tests comparing nested models (*L.Ratio*) and associated p-values. Adjusted *R^2^s* for the ordinary least squares regression models and p-value associated to the multiple regressions are presented. The lowest AICc value for each process, the highest adjusted *R^2^* and the significant differences between models (*p*<0.1) are in bold.

Then, we examined whether each of the three biodiversity components added a significant contribution to the explanation of ecosystem processes using generalized likelihood-ratio tests comparing nested models. The taxonomic component (richness and evenness) never made an additional contribution to the explanation of ecosystem processes since the FI+FD model was not significantly outperformed by any full model (TD+FI+FD) with all 8 indices (Table 1). Conversely, functional identity and functional diversity added a significant contribution for, respectively, 3 and 5 processes. We found that all variance inflation factors were lower than the critical heuristic value of 10 suggesting that collinearity among explanatory variables did not strongly affect our results (see Table S2 for values by predictor).

### Selection of the minimal adequate model and its explanatory power

**Figure 2:**
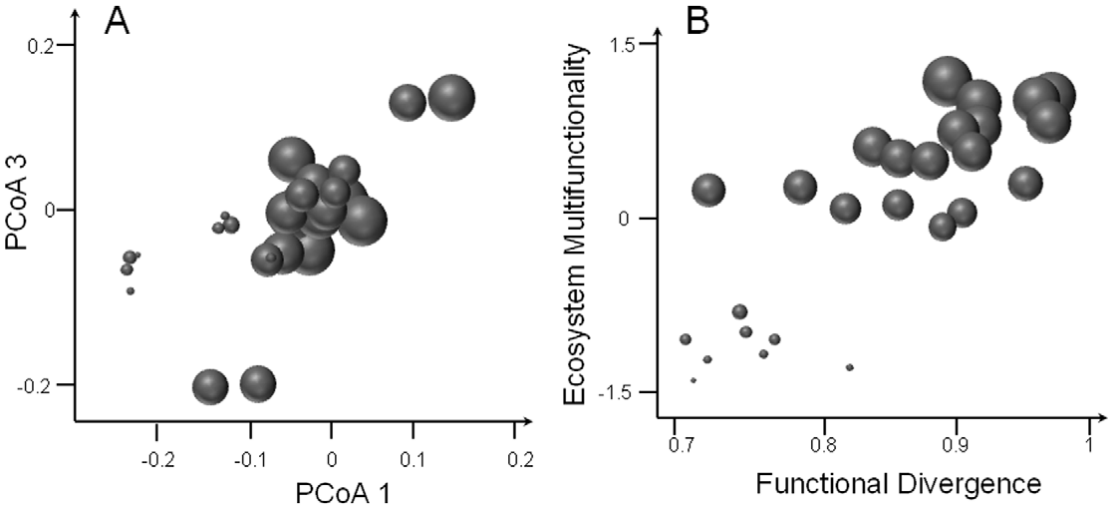
Relationships between community structure and ecosystem multifunctionality. (A) Multifunctionality performance of each community in the functional trait space (first and third axes of the PCoA – PCoA 1 and PCoA 3 respectively). (A) Multifunctionality performance against functional divergence (FDiv). Circle sizes are proportional to performance of communities. See Table 1 for associated statistics.

For each ecosystem process, we performed a multiple regression including the 8 indices as predictive variables with a backward procedure to select the minimal adequate model (Table 2). Biodiversity indices explained significantly, albeit weakly, cotton decomposition (*R* ^2^ = 0.34–0.42) but only functional aspects of community structure were retained in the minimal adequate model, with functional divergence having the main effect (positive) over the two years. For litter decomposition, 69% of the variation was explained by community structure with a combination of three indices: species evenness, functional identity on the second axis and functional divergence, the latter having a positive influence. More interestingly, up to 82% of the variation in productivity was explained by community structure with consistent effects of functional divergence and functional identity (first and second axis) over the two years. Similarly, the functional structure of communities explained nitrogen pool size at 84%, with a predominant positive effect of functional divergence, while species richness was not retained in the final model. Finally, 80% of the level of multifunctionality was explained by only three variables: functional identity (first and third PCoA axes) and functional divergence, with functional divergence having the greatest influence (positive). In other words, the aggregated mean position of the community within functional trait space in combination with functional divergence accurately predicts the level of ecosystem multifunctionality. Figure 2a shows the influence of position in functional space for multifunctionality, with communities having higher values than −0.1 on the first PCoA axis also have higher levels of multifunctionality than the others while communities with low values on both the first and third PCoA axes have a low average multifunctionality values. In addition, all communities with high functional divergence values (>0.85) show high multifunctionality levels (Figure 2b).

**Table 2:** Summary of the minimal adequate models.

	S	E	PC1	PC2	PC3	FRic	FEve	FDiv	*R* ^2^	*p*
Cottondecom 97					−2.0*	−2.1**	−1.9*	3.6***	0.34	0.0140
Cottondecom 98					−2.3**		−4.1***	2.5**	0.42	0.0016
Litterdecom homo		−3.1***		2.4**	−1.7*			2.9***	0.69	<0.0001
Productivity 97	2.3**		3.8***		−2.8***			2.7**	0.82	<0.0001
Productivity 98			1.8*		−2.2**	3.0***		2.4**	0.75	<0.0001
Npool bm 98			3.1***		−2.8**	2.4**		3.4***	0.84	<0.0001
Multifunctionality			3.0***		−3.5***			4.2***	0.80	<0.0001

Results of regressions of ecosystem processes (Cottondecomp: cotton decomposition, Litterdecom 98: litter decomposition in 1998, Productivity: productivity as annual biomass production, Npool bm: nitrogen pool size in aboveground biomass, Multifunctionality: mean performance over all processes) against 8 biodiversity indices (S: species richness, E: species evenness, PC1 PC2 and PC3: aggregated mean trait values along three PCoA axes, FRic: functional richness, FEve: functional evenness, FDiv: functional divergence). *t*-value for each selected variable, adjusted *R* ^2^s for the ordinary least squares regression models and *p*-value associated to the multiple regressions are presented. Explanatory variables (biodiversity indices) were selected using a backward selection procedure starting with a maximal model towards the one containing nothing but significant terms (*p*<0.1).

*p*<0.1,

**p<0.05,

***p<0.01.

**Figure 3:**
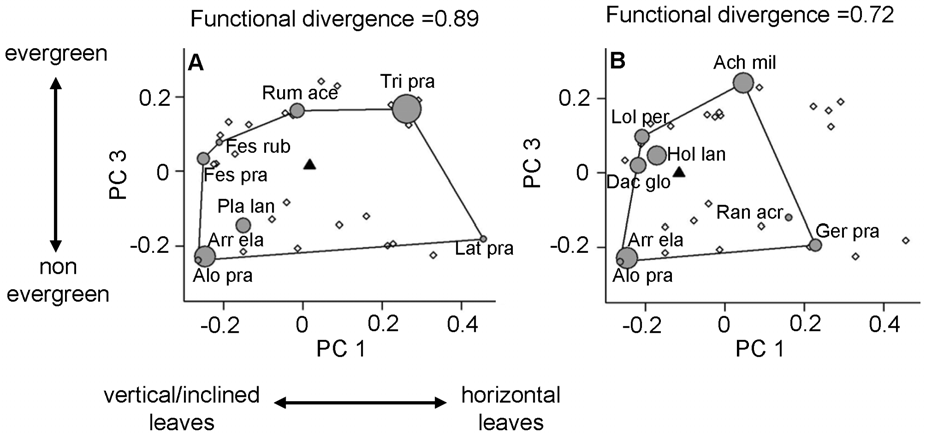
Two species communities represented in functional space with contrasting multifunctionality levels. Two 8-species communities of our experiment with the highest multifunctionality level (a) and the lowest (b). Positions of species are presented in the functional space (first and third PCoA axes). The black triangle labeled “Agg” represents the biomass-weighted mean trait values (aggregated trait) along the two PCoA axes while the lines represent the functional volume occupied by each community. The sizes of grey circles are proportional to species relative abundances. Full species names and trait values can be found in Table S1.

Figure 3 shows two communities containing the same number of species (8) with extreme values along the gradient of multifunctionality level (community *a*>community *b*). In the high functioning community *a*, all the dominant species are specialists (*i.e.* with extreme combinations of traits), which contributes to a high functional divergence value. Community *a* also has a higher mean value on the first PCoA axis of all communities (indicated by the black triangle in Figure 3a). Conversely, the low functioning community *b* has a lower functional divergence value with some dominant species being generalists (i.e. close to the center of the functional space occupied by the community) that are functionally redundant (Figure 3b). This community has also a lower mean value on the first PCoA axis.

### Structural Equation Model

**Figure 4:**
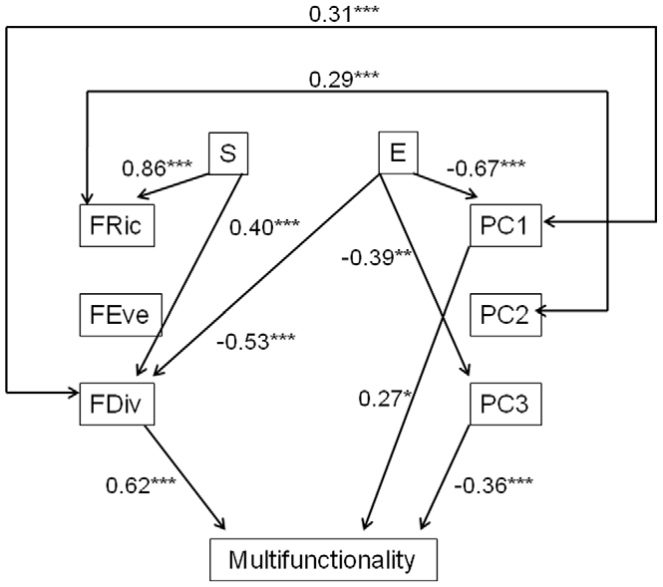
Results of the structural equation model (SEM) linking the multifunctionality of ecosystems to biodiversity indices. (S: species richness, E: evenness in species abundances, PC1 PC2 and PC3: aggregated mean trait values along three PCoA axes, FRic: functional richness, FEve: functional evenness, FDiv: functional divergence.) Numbers next to unidirectional arrows are standardized slopes and those next to bidirectional arrows are correlations. Only significant effects or correlations are shown (* p<0.1, ** p<0.05, *** p<0.01). For detailed statistics and for each process, see Text S2.

Using a structural equation model (SEM) for ecosystem multifunctionality (models for other processes are provided in Text S2), we confirm that taxonomic composition of communities had no direct significant influence on ecosystem multifunctionality (Figure 4); only functional identity (through first and third PCoA axes) and functional divergence had a significant direct effect with functional divergence having the greatest influence (positive). Taxonomic diversity did have a significant influence on the functional structure of communities, but the greatest effect was the positive influence of species richness on functional richness, which had no significant effect on multifunctionality. Functional indices were weakly related between each other and only two correlations were significant and positive (functional divergence and first PCoA axis, functional richness and second PCoA axis). The SEM illustrates that despite the co-linearity between the first PCoA axis and functional divergence, both indices had significant independent effects on multifunctionality.

## Discussion

Our results demonstrate that biodiversity components differ greatly in their influence on ecosystem processes. The taxonomic component, after removing the effects of functional identity and diversity, has no additional effect on processes with consistently low and non significant likelihood-ratio values (Table 1). In addition, species richness and evenness were rarely retained in the minimal adequate model (Table 2) or by the SEM (Figure 4) for their direct influence on ecosystem processes (Text S2). This result can be partly explained by the positive relationship between functional richness and species richness (Figure 4) [40] since communities with more species are more likely to hold a higher diversity of traits and thus perform more functions [47]. Therefore, the additional effect of species richness is likely to be weak after removing the effect of functional richness. Similarly, species evenness has no significant influence on ecosystem processes (except litter decomposition) but it influences the functional structure of communities as revealed by the SEM analysis (Figure 4). We conclude that while the influence of taxonomic structure on ecosystem processes is less important than that of functional identity and diversity, taxonomic composition mediates functional structure. This implies that the taxonomic composition of communities may have indirect effects on ecosystem processes since they are not their proximate, but partly their ultimate, drivers.

While it remains difficult to provide a definite mechanistic explanation for the relationship between functional structure and multifunctionality, existing literature and our own observations may provide some clues. Two of the key functional traits for explaining multifunctionality were leaf phenology (evergreen vs. partly evergreen vs. summergreen) and leaf inclination. There is only very little evidence that increased phenological complementarity can have a positive effect on annual productivity in early successional forb communities, although such an effect might be stronger at low levels of species richness [48]. Evergreen species at our site might have some photosynthetic activity during mild winter days, but biomass production is very low until the onset of spring. Some of the evergreen or partly evergreen species, however, shown an early onset of growth in spring with an early peak in the season (e.g. *Alopecurus pratensis*, *Plantago lanceolata*), while the summergreen species have a tendency to peak later in the year (e.g. *Centaurea jacea*, *Geranium pratense*). Thus, this temporal complementarity of growth might have induced higher productivity with higher functional divergence in leaf phenology. Variability in leaf inclination is known to enhance the photosynthetic light capture of individual tree crowns (e.g. [49]), while in a study of mixed red clover (*Trifolium pratense*) and tall fescue (*Festuca arundinacea*) canopy differences in leaf inclination between the two species increase equality in light partitioning between the taller fescue and shorter clover [50]. In our model plant community, the influence of functional divergence on productivity may be due to temporal and spatial partitioning in light capture via complementarity in phenology and leaf inclination, respectively.

In a previous study on the same site, it has been shown that increasing functional diversity positively influences decomposition rates of plant litter, while species richness had no such effect [31]. These results suggest that this positive effect of functional diversity was due to improved microenvironmental conditions for decomposer fauna, and due to higher litter quality.

With reference to functional identity, increasing dominance of species with more horizontal leaf inclination might enhance productivity by increasing total light capture relative to communities dominated by species with vertical inclination, which might partially explain the influence of PC1 on multifunctionality. Supporting this mechanism, communities dominated by forb species with horizontal leaf inclination also had higher leaf area index than those dominated by grasses with a more vertical inclination. In addition, all nitrogen-fixing legumes planted show a horizontal leaf inclination, partly confounding leaf inclination with N-fixation, the latter being known to positively influence productivity at our site [51]. However, it is unclear how aggregate mean phenology would affect multifunctionality. Perhaps summergreen species are able to grow faster since decreased leaf longevity is associated with increased photosynthetic rates [52]. Short lived leaves also have traits associated with more rapid decomposition rates (e.g. high nutrient content, [27]), which would explain the influence of PC3 on litter decomposition in the minimal adequate regression. The higher nutrient content of summergreen leaves is supported by the negative relationship between PC3 and the amount of nitrogen in biomass in the minimal adequate regression.

The predominance of variables linked to the functional structure of communities over taxonomic variables in predicting ecosystem processes is in accordance with the most recent findings obtained in experiments [20] or with empirical data [9]. Except for decomposition, we show that functional identity and diversity bring independent and additional explanatory power to ecosystem processes with consistently high likelihood-ratio values. Overall, the results suggest that neither functional identity nor functional divergence was more important than the other in explaining ecosystem processes and particularly the multifunctionality. So, contrary to other studies, demonstrating the higher contribution of one component over the others [20] [53], we demonstrate that this differential contribution may depend on the process involved, and when considering multiple processes the magnitude of the two component effects is similar. Thus, to reach high levels of predictability in modelling multiple ecosystem processes, functional identity and diversity components have to be taken into account in a common framework [54].

It has been suggested that, since different species often influence different functions, the level of biodiversity needed to sustain multifunctionality in ecosystems is higher than previously thought [15] [16]. By integrating across four ecosystem processes in assessing the level of community multifunctionality, we show that both functional divergence and functional identity have a predominant role, while species richness has no direct effects (Table 2) and few indirect effects (Figure 4). We suggest that this absence of a species richness effect is partly explained by the relatively high richness values considered in our study (4 to 16 species) while past evidence for positive effects of species richness on ecosystem processes have often been due to the weak performances of monocultures or very species poor communities [2]. Indeed our results are not in contradiction with previous studies demonstrating positive species diversity effects on ecosystem functioning. Rather, they suggest that, except at the extreme low end of species richness gradients, the taxonomic structure of ecological communities is no longer the main driver of ecosystem processes, with the functional structure being the primary determinant.

Our study reconciles two hypotheses that have been alternatively suggested to primarily underpin ecosystem processes: the complementarity and the mass ratio hypotheses. We suggest that a combined effect of functional identity and functional divergence is the most parsimonious explanation for key ecosystem processes. Taken separately, each biodiversity component has weak explanatory power for ecosystem functioning [20] [31] [36]. However, the combined effect of biodiversity components related to the functional structure of communities used in our study consistently reached unprecedented levels of predictive accuracy (up to 84%) whatever the process and for all processes together.

Our finding is crucial since recent work has demonstrated that global gradients in decomposition rates, for example, are primarily driven by plant functional traits rather than climate [27], emphasizing the need for better understanding of the interplay between functional structure of communities and ecosystem functioning. The predominance of functional divergence effects on most of ecosystem processes sheds light on the need to preserve specialist species *sensu* Elton (i.e. those that have a particular combination of traits and perform particular functions in the system). However, since under the combined influence of habitat degradation or global change, we are increasingly losing local specialist species [55] [56], the level of functional diversity held by communities is declining worldwide [57]. Our results show that modifying the functional structure of communities has a strong impact on ecosystem processes and should receive more attention in assessing and countering the global decline of biodiversity.

## Supporting Information

Table S1 Species used in the German BIODEPTH experiment with their traits.
(DOC)

Table S2 Values of the variance inflation factor (VIF) for each biodiversity index (S: species richness, E: species evenness, PC1 PC2 and PC3: aggregated mean trait values along three PCoA axes, FRic: functional richness, FEve: functional evenness, FDiv: functional divergence).
(DOC)

Text S1 Calculation of functional diversity indices.
(DOC)

Text S2 Results from Structural Equation Models (SEM) for each process.
(DOC)
