# Supplementary material for: Functional Structure of Biological Communities Predicts Ecosystem Multifunctionality
Source: PLoS One. 2011 Mar 10;6(3):e17476. doi: 10.1371/journal.pone.0017476 (PMC3053366; doi:10.1371/journal.pone.0017476)
Supplement: Table S1 — Species used in the German BIODEPTH experiment with their traits. (DOC) [file pone.0017476.s001.doc]

Table S1. Species used in the German BIODEPTH experiment with their traits

| Species | Abbreviation | Growth form ^1^ | Leaf size ^2^ | Leaf seasonality | Leaf litter CN ratio ^3^ | SLA (mm^2^ mg^-1^) ^4^ | Predominant leaf orientation ^4^ |
| --- | --- | --- | --- | --- | --- | --- | --- |
| *Achillea millefolium* | Achmil | semirosulata | microphyllous | evergreen | 28.1 | 12.7 | vertical |
| *Alopecurus pratensis* | Alopra | caespitosa | mesophyllous | partly evergreen | 25.2 | 18.8 | inclined |
| *Anthoxanthum odoratum* | Antodo | caespitosa | submicrophyllous | evergreen | 25.2 | 17.8 | vertical |
| *Arrhenatherum elatius* | Arrela | caespitosa | submicrophyllous | partly evergreen | 45.9 | 20.0 | inclined |
| *Bromus hordeaceus* | Brohor | caespitosa | submicrophyllous | summergreen | 101.6 | 16.7 | vertical |
| *Campanula patula* | Campat | semirosulata | nanophyllous | evergreen | 30.6 | 23.3 | horizontal |
| *Centaurea jacea* | Cenjac | semirosulata | mesophyllous | summergreen | 39.0 | 30.6 | vertical |
| *Chrysanthemum leucanthemum* | Chrleu | semirosulata | microphyllous | evergreen | 11.2 | 17.3 | vertical |
| *Crepis biennis* | Crebie | semirosulata | mesophyllous | evergreen | 35.4 | 24.2 | vertical |
| *Cynosurus cristatus* | Cyncri | caespitosa | submicrophyllous | evergreen | 48.8 | 14.6 | vertical |
| *Dactylis glomerata* | Dacglo | caespitosa | mesophyllous | evergreen | 39.3 | 21.2 | inclined |
| *Festuca pratensis* | Fespra | caespitosa | mesophyllous | evergreen | 42.4 | 15.9 | inclined |
| *Festuca rubra* | Fesrub | caespitosa | submicrophyllous | evergreen | 40.3 | 12.1 | inclined |
| *Geranium pratense* | Gerpra | semirosulata | mesophyllous | summergreen | 41.1 | 20.5 | horizontal |
| *Holcus lanatus* | Hollan | caespitosa | submicrophyllous | evergreen | 45.4 | 25.9 | inclined |
| *Knautia arvensis* | Knaarv | semirosulata | mesophyllous | partly evergreen | 33.7 | 21.7 | vertical |
| *Lathyrus pratensis* | Latpra | scandentia | nanophyllous | summergreen | 15.2 | 23.5 | horizontal |
| *Leontodon autumnalis* | Leoaut | rosulata | submicrophyllous | evergreen | 33.8 | 24.5 | vertical |
| *Lolium perenne* | Lolper | caespitosa | submicrophyllous | evergreen | 31.0 | 8.8 | inclined |
| *Lotus corniculatus* | Lotcor | caespitosa | microphyllous | summergreen | 18.0 | 28.7 | horizontal |
| *Lychnis flos-cuculi* | Lycflo | semirosulata | mesophyllous | evergreen | 30.0 | 25.0 | vertical |
| *Phleum pratense* | Phlpra | caespitosa | mesophyllous | evergreen | 41.0 | 20.6 | inclined |
| *Pimpinella major* | Pimmaj | semirosulata | mesophyllous | summergreen | 34.6 | 20.1 | inclined |
| *Plantago lanceolata* | Plalan | rosulata | mesophyllous | partly evergreen | 42.8 | 20.3 | vertical |
| *Ranunculus acris* | Ranacr | semirosulata | submicrophyllous | partly evergreen | 37.7 | 17.8 | horizontal |
| *Rumex acetosa* | Rumace | semirosulata | mesophyllous | evergreen | 15.8 | 18.1 | vertical |
| *Taraxacum officinale* | Taroff | rosulata | mesophyllous | partly evergreen | 20.8 | 22.3 | vertical |
| *Trifolium pratense* | Tripra | semirosulata | microphyllous | evergreen | 20.5 | 22.5 | horizontal |
| *Trifolium repens* | Trirep | reptantia | microphyllous | evergreen | 14.3 | 19.1 | horizontal |
| *Vicia cracca* | Viccra | scandentia | submicrophyllous | summergreen | 16.7 | 27.1 | horizontal |
| *Vicia sepium* | Vicsep | scandentia | submicrophyllous | evergreen | 15.2 | 39.3 | horizontal |

1: following Ellenberg H. & Mueller-Dombois D. (1967). A key to Raunkiaer plant life forms with revised subdivisions. Berichte des Geobotanischen Instituts der ETH, Stiftung Rübel, 37, 56-73.

2: nanophyllous (20–200 mm2), microphyllous (2–6 cm2), submicrophyllous (6–20 cm2) and mesophyllous (20–100 cm2)

3: Scherer-Lorenzen M. (2008). Functional diversity affects decomposition processes in experimental grasslands. Functional Ecology, 22, 547-555.

4: Heisse K., Roscher C., Schumacher J. & Schulze E.D. (2007). Establishment of grassland species in monocultures: different strategies lead to success. Oecologia, 152, 435-447.
