# Supplementary material for: Functional Structure of Biological Communities Predicts Ecosystem Multifunctionality
Source: PLoS One. 2011 Mar 10;6(3):e17476. doi: 10.1371/journal.pone.0017476 (PMC3053366; doi:10.1371/journal.pone.0017476)
Supplement: Table S2 — Values of the variance inflation factor (VIF) for each biodiversity index (S: species richness, E: species evenness, PC1 PC2 and PC3: aggregated mean trait values along three PCoA axes, FRic: functional richness, FEve: functional evenness, FDiv: functional divergence). (DOC) [file pone.0017476.s002.doc]

Table S2. Values of the variance inflation factor (VIF) for each biodiversity index (S: species richness, E: species evenness, PC1 PC2 and PC3: aggregated mean trait values along three PCoA axes, FRic: functional richness, FEve: functional evenness, FDiv: functional divergence).

| S | E | pc1 | pc2 | pc3 | FRic | FEve | FDiv |
| --- | --- | --- | --- | --- | --- | --- | --- |
| 2.6 | 6.1 | 8.1 | 3.1 | 2.7 | 8.8 | 1.1 | 4.4 |
