# Supplementary material for: Functional Structure of Biological Communities Predicts Ecosystem Multifunctionality
Source: PLoS One. 2011 Mar 10;6(3):e17476. doi: 10.1371/journal.pone.0017476 (PMC3053366; doi:10.1371/journal.pone.0017476)
Supplement: Text S1 — Calculation of functional diversity indices. (DOC) [file pone.0017476.s003.doc]

**Functional diversity indices**

T traits and S species are considered. Any species i has T traits of standardized values (x_i1_, x_i2_, …, x_iT_). Relative abundances of species are noted (w_1_, w_2_, …, w_S_), with $\sum_{i=1}^{S} w_{i}=1$. Species relative abundances were quantified with reference to biomass, rather than number of individuals, since biomass, with its direct relationship to metabolism, is more indicative of the functional impact of an individual species within an ecosystem (Grime 1998).

**Functional richness index: FRic**

The first facet of functional diversity is functional richness which represents the amount of functional space filled by the community (Figure 1 [1 and 1']). We estimated functional richness by the volume inside the hull that contains all trait combinations represented in the community, which basically corresponds to a multivariate functional range (FRic) (Cornwe*ll et a*l. 2006; Villeg*er et a*l. 2008). More formally, this measure quantifies the volume inside the minimum convex hull containing all the species belonging to the community. Therefore, functional richness is only influenced by the identity of species (their abundances do not matter) and more particularly by the most extreme species (in terms of functional traits) which delimitate the convex hull.

The convex hull is defined by the following condition: if two species a and b whose traits values are respectively (x_a1_, x_a2_, …, x_aT_) and (x_b1_, x_b2_, …, x_bT_), are inside the convex hull volume, then any hypothetical species with coordinates (Kx_a1_+ (1-K) x_b1_, Kx_a2_+ (1-K) x_b2_,…, Kx_aT_+ (1-K) x_bT_)

for 0≤K≤1 is also in the convex hull volume.

We suggest computing the convex hull volume with the Quickhull algorithm. Basically, the algorithm determines the most extreme points named vertices, links them to build the convex hull and finally calculates the volume inside. The program returns the volume and the identity of the species forming the vertices.

**Functional evenness index: FEve**

The second facet of functional diversity, functional evenness, is defined as the regularity of abundance distribution in the multidimensional functional space. Villéger et al. (2008) proposed an index, named FEve to quantify the regularity with which species abundances fill the functional space (Figure 1 [2 and 2']). This measure includes both the regularity of species distribution and the regularity of their abundances along the “skeleton” of the functional volume occupied. This index is constrained between 0 and 1, and decreases either when functional distances among species are less even or when abundances are less evenly distributed among species, i.e. when the main abundances belong to functionally close species.

In order to transform species distribution in a T dimensional functional space to a distribution on a single-axis, we choose to use the minimum spanning tree (noted MST thereafter). The MST is the tree that links all the points contained in a T dimensional space with the minimum sum of branch lengths. As a first step, for each branch l of the MST the length is divided by the sum of the abundances of the two species linked by the branch: $EW_{l}=\frac{dist(i,j)}{w_{i}+w_{j}}$, where w_i_ is the relative abundance of species i and dist(i,j) is the Euclidean distance between species i and j, the species involved in branch l $dist(i,j)=\sqrt{\sum_{k=1}^{T} \left(x_{ik}-x_{jk}\right)^{2}}$.

Then, for each of these branches, the value of EW_l_ is divided by the sum of EW values for the MST to obtain the partial weighted evenness (PEW) defined as:$PEW_{l}=\frac{EW_{l}}{\sum_{l=1}^{S-1} EW_{l}}$

In the case of perfect regularity of abundance distribution along the MST, all PEW_l_ values will be 1/(S-1). To this aim we compared PEW_l_ values to 1/(S-1).

Finally, our functional evenness index is $FEve=\frac{\sum_{l=1}^{S-1} \min\left(PEW_{l},\frac{1}{S-1}\right)-\frac{1}{S-1}}{1-\frac{1}{S-1}}$

**Computation of the functional divergence index: FDiv**

The third facet of functional diversity, functional divergence, was introduced to quantify whether higher abundances are close to the volume borders (Figure 1 [3 and 3']). The corresponding index, proposed by Villéger et al. (2008), is named FDiv and ranges between 0 and 1. This index approaches zero when highly abundant species are very close to the centre of gravity of the volume occupied and it approaches unity when highly abundant species are very distant from the centre of gravity.

Firstly, the coordinates of the centre of gravity G_V_ (g_1_, g_2_, …, g_T_) of the V vertices are calculated as:

$g_{k}=\frac{1}{V}\sum_{i=1}^{V} x_{ik}$, where x_ik_ is the coordinate of species i on trait k [1, T].

For each of the S species, the Euclidean distance to G_V_ is: $dG_{i}=\sqrt{\sum_{k=1}^{T} \left(x_{ik}-g_{k}\right)^{2}}$, and the mean distance to the centre of gravity is: $\overline{dG}=\frac{1}{S}\sum_{i=1}^{S} dG_{i}$

Then, the sum of abundance-weighted deviances (Δd) and absolute abundance-weighted deviances (Δ|d|) for distances from the centre of gravity are calculated across the species:

$\Delta d=\sum_{i=1}^{S} w_{i}\times(dG_{i}-\overline{dG})$ and $\Delta\left|d\right|=\sum_{i=1}^{S} w_{i}\times\left|dG_{i}-\overline{dG}\right|$

Functional divergence is then calculated as: $FDiv=\frac{\Delta d+\overline{dG}}{\Delta\left|d\right|+\overline{dG}}$

Scripts used to compute functional diversity indices are available online:

<http://www.ecolag.univ-montp2.fr/software>

**References**

Cornwell W.K., Schwilk D.W. & Ackerly D.D. (2006). A trait-based test for habitat filtering: Convex hull volume. Ecology, 87, 1465-1471.

Villeger S., Mason N.W.H. & Mouillot D. (2008). New multidimensional functional diversity indices for a multifaceted framework in functional ecology. Ecology, 89, 2290-2301.
