# Supplementary material for: Functional Structure of Biological Communities Predicts Ecosystem Multifunctionality
Source: PLoS One. 2011 Mar 10;6(3):e17476. doi: 10.1371/journal.pone.0017476 (PMC3053366; doi:10.1371/journal.pone.0017476)
Supplement: Text S2 — Results from Structural Equation Models (SEM) for each process. (DOC) [file pone.0017476.s004.doc]

**SEM Modelling**

**cottondecom98**

Model converged normally after 48 iterations using ML

Minimum Function Chi-square 26.061

Degrees of freedom 3

P-value 0.0000

Chi-square test baseline model:

Minimum Function Chi-square 162.936

Degrees of freedom 35

P-value 0.0000

Full model versus baseline model:

Comparative Fit Index (CFI) 0.820

Tucker-Lewis Index (TLI) -1.103

Loglikelihood and Information Criteria:

Loglikelihood user model (H0) -246.785

Loglikelihood unrestricted model (H1) -233.755

Akaike (AIC) 571.571

Bayesian (BIC) 619.107

Root Mean Square Error of Approximation:

RMSEA 0.555

90 Percent Confidence Interval 0.371 0.759

P-value RMSEA <= 0.05 0.000

Standardized Root Mean Square Residual:

SRMR 0.094

Model estimates:

Estimate Std.err Z-value P(>|z|) Std.lv Std.all

Regressions:

cottondecom97 ~

pc1 -0.289 0.293 -0.988 0.323 -0.289 -0.290

pc2 0.261 0.212 1.229 0.219 0.261 0.253

pc3 -0.253 0.164 -1.547 0.122 -0.253 -0.253

FRic -0.356 0.424 -0.840 0.401 -0.356 -0.354

FEve -0.304 0.147 -2.072 0.038 -0.304 -0.304

FDiv 0.918 0.296 3.098 0.002 0.918 0.936

even -0.157 0.226 -0.696 0.486 -0.157 -0.153

nbsp -0.197 0.357 -0.552 0.581 -0.197 -0.194

pc1 ~

even -0.726 0.143 -5.088 0.000 -0.726 -0.706

nbsp 0.115 0.141 0.812 0.417 0.115 0.113

pc2 ~

even -0.149 0.203 -0.735 0.462 -0.149 -0.150

nbsp 0.083 0.201 0.412 0.680 0.083 0.084

pc3 ~

even -0.441 0.185 -2.388 0.017 -0.441 -0.431

nbsp 0.161 0.183 0.882 0.378 0.161 0.159

FRic ~

even -0.090 0.102 -0.885 0.376 -0.090 -0.089

nbsp 0.856 0.101 8.473 0.000 0.856 0.848

FEve ~

even 0.156 0.209 0.747 0.455 0.156 0.152

nbsp 0.168 0.207 0.813 0.416 0.168 0.166

FDiv ~

even -0.573 0.148 -3.880 0.000 -0.573 -0.548

nbsp 0.374 0.146 2.554 0.011 0.374 0.361

Residual covariances:

FRic ~~

FEve 0.058 0.094 0.619 0.536 0.058 0.059

FRic ~~

FDiv 0.035 0.060 0.580 0.562 0.035 0.034

pc1 ~~

FRic -0.004 0.047 -0.081 0.935 -0.004 -0.004

pc2 ~~

FRic 0.297 0.108 2.758 0.006 0.297 0.310

pc3 ~~

FRic 0.051 0.063 0.804 0.421 0.051 0.051

FEve ~~

FDiv 0.069 0.136 0.510 0.610 0.069 0.068

pc1 ~~

FEve -0.051 0.131 -0.392 0.695 -0.051 -0.051

pc2 ~~

FEve -0.015 0.184 -0.083 0.934 -0.015 -0.016

pc3 ~~

FEve -0.056 0.169 -0.334 0.738 -0.056 -0.057

pc1 ~~

FDiv 0.303 0.110 2.770 0.006 0.303 0.298

pc2 ~~

FDiv -0.095 0.100 -0.947 0.344 -0.095 -0.097

pc3 ~~

FDiv 0.016 0.088 0.178 0.859 0.016 0.015

Residual variances:

cottondecom97 0.480 0.136 3.536 0.000 0.480 0.483

pc1 0.445 0.126 3.536 0.000 0.445 0.445

pc2 0.899 0.254 3.536 0.000 0.899 0.964

pc3 0.744 0.210 3.536 0.000 0.744 0.752

FRic 0.228 0.064 3.559 0.000 0.228 0.232

FEve 0.956 0.270 3.536 0.000 0.956 0.963

FDiv 0.477 0.134 3.569 0.000 0.477 0.461

**cottondecom98**

Model converged normally after 44 iterations using ML

Minimum Function Chi-square 27.825

Degrees of freedom 3

P-value 0.0000

Chi-square test baseline model:

Minimum Function Chi-square 168.259

Degrees of freedom 35

P-value 0.0000

Full model versus baseline model:

Comparative Fit Index (CFI) 0.814

Tucker-Lewis Index (TLI) -1.173

Loglikelihood and Information Criteria:

Loglikelihood user model (H0) -256.598

Loglikelihood unrestricted model (H1) -242.685

Akaike (AIC) 591.195

Bayesian (BIC) 640.261

Root Mean Square Error of Approximation:

RMSEA 0.564

90 Percent Confidence Interval 0.384 0.764

P-value RMSEA <= 0.05 0.000

Standardized Root Mean Square Residual:

SRMR 0.098

Model estimates:

Estimate Std.err Z-value P(>|z|) Std.lv Std.all

Regressions:

cottondecom98 ~

pc1 -0.059 0.280 -0.211 0.833 -0.059 -0.058

pc2 -0.092 0.193 -0.479 0.632 -0.092 -0.090

pc3 -0.421 0.157 -2.688 0.007 -0.421 -0.409

FRic 0.392 0.406 0.966 0.334 0.392 0.381

FEve -0.668 0.144 -4.625 0.000 -0.668 -0.647

FDiv 0.382 0.289 1.322 0.186 0.382 0.377

even -0.045 0.205 -0.220 0.826 -0.045 -0.044

nbsp -0.215 0.340 -0.633 0.527 -0.215 -0.209

pc1 ~

even -0.677 0.138 -4.913 0.000 -0.677 -0.677

nbsp 0.157 0.138 1.142 0.253 0.157 0.157

pc2 ~

even -0.069 0.198 -0.348 0.728 -0.069 -0.069

nbsp 0.153 0.198 0.772 0.440 0.153 0.153

pc3 ~

even -0.390 0.176 -2.213 0.027 -0.390 -0.390

nbsp 0.205 0.176 1.161 0.246 0.205 0.205

FRic ~

even -0.084 0.094 -0.891 0.373 -0.084 -0.084

nbsp 0.862 0.094 9.140 0.000 0.862 0.861

FEve ~

even 0.148 0.196 0.754 0.451 0.148 0.149

nbsp 0.161 0.196 0.820 0.412 0.161 0.162

FDiv ~

even -0.539 0.140 -3.849 0.000 -0.539 -0.530

nbsp 0.404 0.140 2.883 0.004 0.404 0.397

Residual covariances:

FRic ~~

FEve 0.057 0.087 0.655 0.512 0.057 0.059

FRic ~~

FDiv 0.039 0.056 0.696 0.486 0.039 0.040

pc1 ~~

FRic 0.003 0.047 0.057 0.955 0.003 0.003

pc2 ~~

FRic 0.280 0.103 2.728 0.006 0.280 0.291

pc3 ~~

FRic 0.031 0.061 0.508 0.611 0.031 0.032

FEve ~~

FDiv 0.064 0.129 0.494 0.622 0.064 0.065

pc1 ~~

FEve -0.053 0.127 -0.417 0.677 -0.053 -0.055

pc2 ~~

FEve -0.013 0.180 -0.070 0.944 -0.013 -0.013

pc3 ~~

FEve -0.054 0.162 -0.332 0.740 -0.054 -0.056

pc1 ~~

FDiv 0.306 0.108 2.845 0.004 0.306 0.313

pc2 ~~

FDiv -0.088 0.098 -0.903 0.367 -0.088 -0.090

pc3 ~~

FDiv 0.023 0.085 0.269 0.788 0.023 0.023

Residual variances:

cottondecom98 0.464 0.129 3.606 0.000 0.464 0.455

pc1 0.452 0.125 3.606 0.000 0.452 0.470

pc2 0.930 0.258 3.606 0.000 0.930 0.967

pc3 0.741 0.206 3.606 0.000 0.741 0.771

FRic 0.212 0.059 3.614 0.000 0.212 0.220

FEve 0.919 0.255 3.606 0.000 0.919 0.962

FDiv 0.467 0.128 3.637 0.000 0.467 0.470

**wooddecomp**

Model converged normally after 54 iterations using ML

Minimum Function Chi-square 28.585

Degrees of freedom 3

P-value 0.0000

Chi-square test baseline model:

Minimum Function Chi-square 143.882

Degrees of freedom 35

P-value 0.0000

Full model versus baseline model:

Comparative Fit Index (CFI) 0.765

Tucker-Lewis Index (TLI) -1.741

Loglikelihood and Information Criteria:

Loglikelihood user model (H0) -221.889

Loglikelihood unrestricted model (H1) -207.596

Akaike (AIC) 521.778

Bayesian (BIC) 564.329

Root Mean Square Error of Approximation:

RMSEA 0.623

90 Percent Confidence Interval 0.427 0.840

P-value RMSEA <= 0.05 0.000

Standardized Root Mean Square Residual:

SRMR 0.118

Model estimates:

Estimate Std.err Z-value P(>|z|) Std.lv Std.all

Regressions:

wooddecomp ~

pc1 0.177 0.352 0.505 0.614 0.177 0.172

pc2 -0.067 0.258 -0.260 0.795 -0.067 -0.060

pc3 -0.644 0.229 -2.811 0.005 -0.644 -0.525

FRic 0.444 0.553 0.803 0.422 0.444 0.393

FEve -0.123 0.178 -0.691 0.490 -0.123 -0.118

FDiv 0.392 0.356 1.102 0.271 0.392 0.380

even 0.061 0.287 0.211 0.833 0.061 0.056

nbsp -0.601 0.442 -1.359 0.174 -0.601 -0.529

pc1 ~

even -0.750 0.148 -5.084 0.000 -0.750 -0.717

nbsp 0.164 0.155 1.061 0.288 0.164 0.150

pc2 ~

even -0.172 0.200 -0.864 0.388 -0.172 -0.179

nbsp 0.227 0.209 1.083 0.279 0.227 0.225

pc3 ~

even -0.415 0.169 -2.454 0.014 -0.415 -0.470

nbsp 0.041 0.177 0.232 0.817 0.041 0.044

FRic ~

even -0.081 0.098 -0.822 0.411 -0.081 -0.085

nbsp 0.864 0.103 8.354 0.000 0.864 0.860

FEve ~

even 0.157 0.222 0.708 0.479 0.157 0.151

nbsp 0.207 0.234 0.886 0.375 0.207 0.189

FDiv ~

even -0.585 0.158 -3.689 0.000 -0.585 -0.558

nbsp 0.386 0.166 2.319 0.020 0.386 0.351

Residual covariances:

FRic ~~

FEve 0.057 0.101 0.562 0.574 0.057 0.055

FRic ~~

FDiv 0.109 0.071 1.527 0.127 0.109 0.105

pc1 ~~

FRic 0.088 0.058 1.522 0.128 0.088 0.085

pc2 ~~

FRic 0.237 0.100 2.385 0.017 0.237 0.250

pc3 ~~

FRic -0.047 0.060 -0.776 0.438 -0.047 -0.054

FEve ~~

FDiv 0.096 0.163 0.588 0.557 0.096 0.085

pc1 ~~

FEve -0.041 0.150 -0.275 0.783 -0.041 -0.037

pc2 ~~

FEve -0.064 0.203 -0.312 0.755 -0.064 -0.062

pc3 ~~

FEve -0.042 0.172 -0.247 0.805 -0.042 -0.045

pc1 ~~

FDiv 0.331 0.127 2.596 0.009 0.331 0.293

pc2 ~~

FDiv -0.097 0.111 -0.873 0.383 -0.097 -0.094

pc3 ~~

FDiv 0.003 0.091 0.032 0.975 0.003 0.003

Residual variances:

wooddecomp 0.685 0.207 3.317 0.001 0.685 0.569

pc1 0.468 0.141 3.317 0.001 0.468 0.415

pc2 0.856 0.258 3.317 0.001 0.856 0.899

pc3 0.614 0.185 3.317 0.001 0.614 0.767

FRic 0.209 0.061 3.426 0.001 0.209 0.221

FEve 1.064 0.321 3.317 0.001 1.064 0.954

FDiv 0.540 0.161 3.346 0.001 0.540 0.478

**litterdecomhomo**

Model converged normally after 47 iterations using ML

Minimum Function Chi-square 27.825

Degrees of freedom 3

P-value 0.0000

Chi-square test baseline model:

Minimum Function Chi-square 186.514

Degrees of freedom 35

P-value 0.0000

Full model versus baseline model:

Comparative Fit Index (CFI) 0.836

Tucker-Lewis Index (TLI) -0.912

Loglikelihood and Information Criteria:

Loglikelihood user model (H0) -247.470

Loglikelihood unrestricted model (H1) -233.558

Akaike (AIC) 572.941

Bayesian (BIC) 622.007

Root Mean Square Error of Approximation:

RMSEA 0.564

90 Percent Confidence Interval 0.384 0.764

P-value RMSEA <= 0.05 0.000

Standardized Root Mean Square Residual:

SRMR 0.099

Model estimates:

Estimate Std.err Z-value P(>|z|) Std.lv Std.all

Regressions:

litterdecomhomo ~

pc1 0.090 0.197 0.456 0.648 0.090 0.090

pc2 0.249 0.136 1.838 0.066 0.249 0.250

pc3 -0.281 0.110 -2.553 0.011 -0.281 -0.283

FRic 0.047 0.286 0.166 0.868 0.047 0.048

FEve 0.017 0.102 0.172 0.864 0.017 0.017

FDiv 0.565 0.203 2.781 0.005 0.565 0.577

even -0.387 0.144 -2.685 0.007 -0.387 -0.389

nbsp -0.194 0.239 -0.813 0.416 -0.194 -0.195

pc1 ~

even -0.677 0.138 -4.913 0.000 -0.677 -0.677

nbsp 0.157 0.138 1.142 0.253 0.157 0.157

pc2 ~

even -0.069 0.198 -0.348 0.728 -0.069 -0.069

nbsp 0.153 0.198 0.772 0.440 0.153 0.153

pc3 ~

even -0.390 0.176 -2.213 0.027 -0.390 -0.390

nbsp 0.205 0.176 1.161 0.246 0.205 0.205

FRic ~

even -0.084 0.094 -0.891 0.373 -0.084 -0.084

nbsp 0.862 0.094 9.140 0.000 0.862 0.861

FEve ~

even 0.148 0.196 0.754 0.451 0.148 0.149

nbsp 0.161 0.196 0.820 0.412 0.161 0.162

FDiv ~

even -0.539 0.140 -3.849 0.000 -0.539 -0.530

nbsp 0.404 0.140 2.883 0.004 0.404 0.397

Residual covariances:

FRic ~~

FEve 0.057 0.087 0.655 0.512 0.057 0.059

FRic ~~

FDiv 0.039 0.056 0.696 0.486 0.039 0.040

pc1 ~~

FRic 0.003 0.047 0.057 0.954 0.003 0.003

pc2 ~~

FRic 0.280 0.103 2.728 0.006 0.280 0.291

pc3 ~~

FRic 0.031 0.061 0.508 0.611 0.031 0.032

FEve ~~

FDiv 0.064 0.129 0.494 0.622 0.064 0.065

pc1 ~~

FEve -0.053 0.127 -0.417 0.677 -0.053 -0.055

pc2 ~~

FEve -0.013 0.180 -0.070 0.944 -0.013 -0.013

pc3 ~~

FEve -0.054 0.162 -0.333 0.739 -0.054 -0.056

pc1 ~~

FDiv 0.306 0.108 2.845 0.004 0.306 0.313

pc2 ~~

FDiv -0.088 0.098 -0.903 0.367 -0.088 -0.090

pc3 ~~

FDiv 0.023 0.085 0.269 0.788 0.023 0.023

Residual variances:

litterdecomhomo 0.230 0.064 3.606 0.000 0.230 0.241

pc1 0.452 0.125 3.606 0.000 0.452 0.470

pc2 0.930 0.258 3.606 0.000 0.930 0.967

pc3 0.741 0.206 3.606 0.000 0.741 0.771

FRic 0.212 0.059 3.614 0.000 0.212 0.220

FEve 0.919 0.255 3.606 0.000 0.919 0.962

FDiv 0.467 0.128 3.637 0.000 0.467 0.470

**Prod97**

Model converged normally after 49 iterations using ML

Minimum Function Chi-square 27.825

Degrees of freedom 3

P-value 0.0000

Chi-square test baseline model:

Minimum Function Chi-square 200.481

Degrees of freedom 35

P-value 0.0000

Full model versus baseline model:

Comparative Fit Index (CFI) 0.850

Tucker-Lewis Index (TLI) -0.750

Loglikelihood and Information Criteria:

Loglikelihood user model (H0) -240.487

Loglikelihood unrestricted model (H1) -226.574

Akaike (AIC) 558.973

Bayesian (BIC) 608.039

Root Mean Square Error of Approximation:

RMSEA 0.564

90 Percent Confidence Interval 0.384 0.764

P-value RMSEA <= 0.05 0.000

Standardized Root Mean Square Residual:

SRMR 0.113

Model estimates:

Estimate Std.err Z-value P(>|z|) Std.lv Std.all

Regressions:

prod97 ~

pc1 0.445 0.151 2.948 0.003 0.445 0.425

pc2 0.142 0.104 1.370 0.171 0.142 0.136

pc3 -0.261 0.084 -3.099 0.002 -0.261 -0.250

FRic -0.159 0.218 -0.730 0.466 -0.159 -0.152

FEve -0.030 0.078 -0.390 0.696 -0.030 -0.029

FDiv 0.513 0.155 3.303 0.001 0.513 0.499

even -0.029 0.110 -0.265 0.791 -0.029 -0.028

nbsp 0.338 0.183 1.851 0.064 0.338 0.323

pc1 ~

even -0.677 0.138 -4.913 0.000 -0.677 -0.677

nbsp 0.157 0.138 1.142 0.253 0.157 0.157

pc2 ~

even -0.069 0.198 -0.348 0.728 -0.069 -0.069

nbsp 0.153 0.198 0.772 0.440 0.153 0.153

pc3 ~

even -0.390 0.176 -2.213 0.027 -0.390 -0.390

nbsp 0.205 0.176 1.161 0.246 0.205 0.205

FRic ~

even -0.084 0.094 -0.891 0.373 -0.084 -0.084

nbsp 0.862 0.094 9.140 0.000 0.862 0.861

FEve ~

even 0.148 0.196 0.754 0.451 0.148 0.149

nbsp 0.161 0.196 0.820 0.412 0.161 0.162

FDiv ~

even -0.539 0.140 -3.849 0.000 -0.539 -0.530

nbsp 0.404 0.140 2.883 0.004 0.404 0.397

Residual covariances:

FRic ~~

FEve 0.057 0.087 0.655 0.512 0.057 0.059

FRic ~~

FDiv 0.039 0.056 0.696 0.486 0.039 0.040

pc1 ~~

FRic 0.003 0.047 0.057 0.955 0.003 0.003

pc2 ~~

FRic 0.280 0.103 2.728 0.006 0.280 0.291

pc3 ~~

FRic 0.031 0.061 0.508 0.611 0.031 0.032

FEve ~~

FDiv 0.064 0.129 0.494 0.622 0.064 0.065

pc1 ~~

FEve -0.053 0.127 -0.417 0.677 -0.053 -0.055

pc2 ~~

FEve -0.013 0.180 -0.070 0.944 -0.013 -0.013

pc3 ~~

FEve -0.054 0.162 -0.333 0.739 -0.054 -0.056

pc1 ~~

FDiv 0.306 0.108 2.845 0.004 0.306 0.313

pc2 ~~

FDiv -0.088 0.098 -0.903 0.367 -0.088 -0.090

pc3 ~~

FDiv 0.023 0.085 0.269 0.788 0.023 0.023

Residual variances:

prod97 0.134 0.037 3.606 0.000 0.134 0.128

pc1 0.452 0.125 3.606 0.000 0.452 0.470

pc2 0.930 0.258 3.606 0.000 0.930 0.967

pc3 0.741 0.206 3.606 0.000 0.741 0.771

FRic 0.212 0.059 3.614 0.000 0.212 0.220

FEve 0.919 0.255 3.606 0.000 0.919 0.962

FDiv 0.467 0.128 3.637 0.000 0.467 0.470

**Prod98**

Model converged normally after 48 iterations using ML

Minimum Function Chi-square 27.825

Degrees of freedom 3

P-value 0.0000

Chi-square test baseline model:

Minimum Function Chi-square 191.782

Degrees of freedom 35

P-value 0.0000

Full model versus baseline model:

Comparative Fit Index (CFI) 0.842

Tucker-Lewis Index (TLI) -0.847

Loglikelihood and Information Criteria:

Loglikelihood user model (H0) -244.836

Loglikelihood unrestricted model (H1) -230.924

Akaike (AIC) 567.673

Bayesian (BIC) 616.739

Root Mean Square Error of Approximation:

RMSEA 0.564

90 Percent Confidence Interval 0.384 0.764

P-value RMSEA <= 0.05 0.000

Standardized Root Mean Square Residual:

SRMR 0.114

Model estimates:

Estimate Std.err Z-value P(>|z|) Std.lv Std.all

Regressions:

prod98 ~

pc1 0.447 0.178 2.508 0.012 0.447 0.418

pc2 -0.173 0.123 -1.412 0.158 -0.173 -0.162

pc3 -0.353 0.100 -3.540 0.000 -0.353 -0.330

FRic 0.710 0.258 2.753 0.006 0.710 0.664

FEve -0.039 0.092 -0.428 0.669 -0.039 -0.037

FDiv 0.437 0.184 2.380 0.017 0.437 0.415

even 0.069 0.130 0.532 0.595 0.069 0.065

nbsp -0.274 0.216 -1.266 0.205 -0.274 -0.256

pc1 ~

even -0.677 0.138 -4.913 0.000 -0.677 -0.677

nbsp 0.157 0.138 1.142 0.253 0.157 0.157

pc2 ~

even -0.069 0.198 -0.348 0.728 -0.069 -0.069

nbsp 0.153 0.198 0.772 0.440 0.153 0.153

pc3 ~

even -0.390 0.176 -2.213 0.027 -0.390 -0.390

nbsp 0.205 0.176 1.161 0.246 0.205 0.205

FRic ~

even -0.084 0.094 -0.891 0.373 -0.084 -0.084

nbsp 0.862 0.094 9.140 0.000 0.862 0.861

FEve ~

even 0.148 0.196 0.754 0.451 0.148 0.149

nbsp 0.161 0.196 0.820 0.412 0.161 0.162

FDiv ~

even -0.539 0.140 -3.849 0.000 -0.539 -0.530

nbsp 0.404 0.140 2.883 0.004 0.404 0.397

Residual covariances:

FRic ~~

FEve 0.057 0.087 0.655 0.512 0.057 0.059

FRic ~~

FDiv 0.039 0.056 0.696 0.486 0.039 0.040

pc1 ~~

FRic 0.003 0.047 0.057 0.955 0.003 0.003

pc2 ~~

FRic 0.280 0.103 2.728 0.006 0.280 0.291

pc3 ~~

FRic 0.031 0.061 0.508 0.611 0.031 0.032

FEve ~~

FDiv 0.064 0.129 0.494 0.622 0.064 0.065

pc1 ~~

FEve -0.053 0.127 -0.417 0.677 -0.053 -0.055

pc2 ~~

FEve -0.013 0.180 -0.070 0.944 -0.013 -0.013

pc3 ~~

FEve -0.054 0.162 -0.333 0.739 -0.054 -0.056

pc1 ~~

FDiv 0.306 0.108 2.845 0.004 0.306 0.313

pc2 ~~

FDiv -0.088 0.098 -0.903 0.367 -0.088 -0.090

pc3 ~~

FDiv 0.023 0.085 0.269 0.788 0.023 0.023

Residual variances:

prod98 0.188 0.052 3.606 0.000 0.188 0.171

pc1 0.452 0.125 3.606 0.000 0.452 0.470

pc2 0.930 0.258 3.606 0.000 0.930 0.967

pc3 0.741 0.206 3.606 0.000 0.741 0.771

FRic 0.212 0.059 3.614 0.000 0.212 0.220

FEve 0.919 0.255 3.606 0.000 0.919 0.962

FDiv 0.467 0.128 3.637 0.000 0.467 0.470

**Npoolbm98**

Model converged normally after 51 iterations using ML

Minimum Function Chi-square 27.825

Degrees of freedom 3

P-value 0.0000

Chi-square test baseline model:

Minimum Function Chi-square 204.299

Degrees of freedom 35

P-value 0.0000

Full model versus baseline model:

Comparative Fit Index (CFI) 0.853

Tucker-Lewis Index (TLI) -0.711

Loglikelihood and Information Criteria:

Loglikelihood user model (H0) -238.578

Loglikelihood unrestricted model (H1) -224.665

Akaike (AIC) 555.156

Bayesian (BIC) 604.221

Root Mean Square Error of Approximation:

RMSEA 0.564

90 Percent Confidence Interval 0.384 0.764

P-value RMSEA <= 0.05 0.000

Standardized Root Mean Square Residual:

SRMR 0.125

Model estimates:

Estimate Std.err Z-value P(>|z|) Std.lv Std.all

Regressions:

Npoolbm98 ~

pc1 0.591 0.140 4.215 0.000 0.591 0.530

pc2 -0.177 0.096 -1.834 0.067 -0.177 -0.159

pc3 -0.366 0.078 -4.675 0.000 -0.366 -0.329

FRic 0.486 0.203 2.396 0.017 0.486 0.436

FEve 0.040 0.072 0.550 0.582 0.040 0.036

FDiv 0.453 0.144 3.137 0.002 0.453 0.413

even 0.014 0.102 0.141 0.888 0.014 0.013

nbsp -0.178 0.170 -1.046 0.295 -0.178 -0.160

pc1 ~

even -0.677 0.138 -4.913 0.000 -0.677 -0.677

nbsp 0.157 0.138 1.142 0.253 0.157 0.157

pc2 ~

even -0.069 0.198 -0.348 0.728 -0.069 -0.069

nbsp 0.153 0.198 0.772 0.440 0.153 0.153

pc3 ~

even -0.390 0.176 -2.213 0.027 -0.390 -0.390

nbsp 0.205 0.176 1.161 0.246 0.205 0.205

FRic ~

even -0.084 0.094 -0.891 0.373 -0.084 -0.084

nbsp 0.862 0.094 9.140 0.000 0.862 0.861

FEve ~

even 0.148 0.196 0.754 0.451 0.148 0.149

nbsp 0.161 0.196 0.820 0.412 0.161 0.162

FDiv ~

even -0.539 0.140 -3.849 0.000 -0.539 -0.530

nbsp 0.404 0.140 2.883 0.004 0.404 0.397

Residual covariances:

FRic ~~

FEve 0.057 0.087 0.655 0.512 0.057 0.059

FRic ~~

FDiv 0.039 0.056 0.696 0.486 0.039 0.040

pc1 ~~

FRic 0.003 0.047 0.057 0.955 0.003 0.003

pc2 ~~

FRic 0.280 0.103 2.728 0.006 0.280 0.291

pc3 ~~

FRic 0.031 0.061 0.508 0.611 0.031 0.032

FEve ~~

FDiv 0.064 0.129 0.494 0.622 0.064 0.065

pc1 ~~

FEve -0.053 0.127 -0.417 0.677 -0.053 -0.055

pc2 ~~

FEve -0.013 0.180 -0.070 0.944 -0.013 -0.013

pc3 ~~

FEve -0.054 0.162 -0.333 0.739 -0.054 -0.056

pc1 ~~

FDiv 0.306 0.108 2.845 0.004 0.306 0.313

pc2 ~~

FDiv -0.088 0.098 -0.903 0.367 -0.088 -0.090

pc3 ~~

FDiv 0.023 0.085 0.269 0.788 0.023 0.023

Residual variances:

Npoolbm98 0.116 0.032 3.606 0.000 0.116 0.097

pc1 0.452 0.125 3.606 0.000 0.452 0.470

pc2 0.930 0.258 3.606 0.000 0.930 0.967

pc3 0.741 0.206 3.606 0.000 0.741 0.771

FRic 0.212 0.059 3.614 0.000 0.212 0.220

FEve 0.919 0.255 3.606 0.000 0.919 0.962

FDiv 0.467 0.128 3.637 0.000 0.467 0.470

**Multifunctionality**

Model converged normally after 50 iterations using ML

Minimum Function Chi-square 27.825

Degrees of freedom 3

P-value 0.0000

Chi-square test baseline model:

Minimum Function Chi-square 195.467

Degrees of freedom 35

P-value 0.0000

Full model versus baseline model:

Comparative Fit Index (CFI) 0.845

Tucker-Lewis Index (TLI) -0.805

Loglikelihood and Information Criteria:

Loglikelihood user model (H0) -242.994

Loglikelihood unrestricted model (H1) -229.081

Akaike (AIC) 563.988

Bayesian (BIC) 613.054

Root Mean Square Error of Approximation:

RMSEA 0.564

90 Percent Confidence Interval 0.384 0.764

P-value RMSEA <= 0.05 0.000

Standardized Root Mean Square Residual:

SRMR 0.112

Model estimates:

Estimate Std.err Z-value P(>|z|) Std.lv Std.all

Regressions:

Multi ~

pc1 0.283 0.166 1.702 0.089 0.283 0.267

pc2 0.052 0.114 0.460 0.646 0.052 0.050

pc3 -0.385 0.093 -4.149 0.000 -0.385 -0.364

FRic 0.240 0.240 0.998 0.318 0.240 0.227

FEve -0.140 0.086 -1.636 0.102 -0.140 -0.132

FDiv 0.653 0.171 3.814 0.000 0.653 0.627

even -0.132 0.121 -1.088 0.277 -0.132 -0.125

nbsp -0.156 0.201 -0.773 0.440 -0.156 -0.147

pc1 ~

even -0.677 0.138 -4.913 0.000 -0.677 -0.677

nbsp 0.157 0.138 1.142 0.253 0.157 0.157

pc2 ~

even -0.069 0.198 -0.348 0.728 -0.069 -0.069

nbsp 0.153 0.198 0.772 0.440 0.153 0.153

pc3 ~

even -0.390 0.176 -2.213 0.027 -0.390 -0.390

nbsp 0.205 0.176 1.161 0.246 0.205 0.205

FRic ~

even -0.084 0.094 -0.891 0.373 -0.084 -0.084

nbsp 0.862 0.094 9.140 0.000 0.862 0.861

FEve ~

even 0.148 0.196 0.754 0.451 0.148 0.149

nbsp 0.161 0.196 0.820 0.412 0.161 0.162

FDiv ~

even -0.539 0.140 -3.849 0.000 -0.539 -0.530

nbsp 0.404 0.140 2.883 0.004 0.404 0.397

Residual covariances:

FRic ~~

FEve 0.057 0.087 0.655 0.512 0.057 0.059

FRic ~~

FDiv 0.039 0.056 0.696 0.486 0.039 0.040

pc1 ~~

FRic 0.003 0.047 0.057 0.954 0.003 0.003

pc2 ~~

FRic 0.280 0.103 2.728 0.006 0.280 0.291

pc3 ~~

FRic 0.031 0.061 0.508 0.611 0.031 0.032

FEve ~~

FDiv 0.064 0.129 0.494 0.622 0.064 0.065

pc1 ~~

FEve -0.053 0.127 -0.417 0.677 -0.053 -0.055

pc2 ~~

FEve -0.013 0.180 -0.070 0.944 -0.013 -0.013

pc3 ~~

FEve -0.054 0.162 -0.333 0.739 -0.054 -0.056

pc1 ~~

FDiv 0.306 0.108 2.845 0.004 0.306 0.313

pc2 ~~

FDiv -0.088 0.098 -0.903 0.367 -0.088 -0.090

pc3 ~~

FDiv 0.023 0.085 0.269 0.788 0.023 0.023

Residual variances:

Multi 0.163 0.045 3.606 0.000 0.163 0.151

pc1 0.452 0.125 3.606 0.000 0.452 0.470

pc2 0.930 0.258 3.606 0.000 0.930 0.967

pc3 0.741 0.206 3.606 0.000 0.741 0.771

FRic 0.212 0.059 3.614 0.000 0.212 0.220

FEve 0.919 0.255 3.606 0.000 0.919 0.962

FDiv 0.467 0.128 3.637 0.000 0.467 0.470
